# How Properties of Kenaf Fibers from Burkina Faso Contribute to the Reinforcement of Earth Blocks

**DOI:** 10.3390/ma8052332

**Published:** 2015-04-30

**Authors:** Younoussa Millogo, Jean-Emmanuel Aubert, Erwan Hamard, Jean-Claude Morel

**Affiliations:** 1 Unité de Formation et de Recherche en Sciences et Techniques (UFR/ST), Université Polytechnique de Bobo-Dioulasso, 01 BP 1091 Bobo 01, Burkina Faso; E-Mail: millogokadi@gmail.com; 2 Laboratoire de Chimie Moléculaire et de Matériaux (LCMM), UFR/Sciences Exactes et Appliquées, Université de Ouagadougou, 03 BP 7021 Ouagadougou 03, Burkina Faso; 3 Université de Toulouse, UPS, INSA, LMDC (Laboratoire Matériaux et Durabilité des Constructions), 135 avenue de Rangueil, F-31077 Toulouse cedex 4, France; E-Mail: jean-emmanuel.aubert@univ-tlse3.fr; 4 Institut Français des Sciences et Technologies des Transports, de l’Aménagement et des Réseaux, Département Matériaux, GPEM, route de Bouaye, 44344 Bouguenais, CS4, France; E-Mail: erwan.hamard@ifsttar.fr; 5 Ecole Nationale des Travaux Publics de l’Etat, Université de Lyon CNRS-LTDS, UMR 5513, LGCB, 3 rue Maurice Audin, Vaulx-en-Velin cedex, F-69120, France

**Keywords:** kenaf fibers, Earth blocks, physicochemical characteristics, mechanical properties, Burkina Faso

## Abstract

Physicochemical characteristics of Hibiscus cannabinus (kenaf) fibers from Burkina Faso were studied using X-ray diffraction (XRD), infrared spectroscopy, thermal gravimetric analysis (TGA), chemical analysis and video microscopy. Kenaf fibers (3 cm long) were used to reinforce earth blocks, and the mechanical properties of reinforced blocks, with fiber contents ranging from 0.2 to 0.8 wt%, were investigated. The fibers were mainly composed of cellulose type I (70.4 wt%), hemicelluloses (18.9 wt%) and lignin (3 wt%) and were characterized by high tensile strength (1 ± 0.25 GPa) and Young’s modulus (136 ± 25 GPa), linked to their high cellulose content. The incorporation of short fibers of kenaf reduced the propagation of cracks in the blocks, through the good adherence of fibers to the clay matrix, and therefore improved their mechanical properties. Fiber incorporation was particularly beneficial for the bending strength of earth blocks because it reinforces these blocks after the failure of soil matrix observed for unreinforced blocks. Blocks reinforced with such fibers had a ductile tensile behavior that made them better building materials for masonry structures than unreinforced blocks.

## 1. Introduction

Hibiscus cannabinus, or kenaf, is a plant of the Malvaceae family that grows in tropical and sub-tropical areas. It is an annual herbaceous plant (or, rarely, a short-lived perennial) growing to 1.5 to 3.5 m tall, with a woody base. The stems are 1 cm to 2 cm in diameter and often but not always branched. The leaves are 10–15 cm long and variable in shape, with leaves near the base of the stems being deeply lobed with 3–7 lobes, while leaves near the top of the stem are weakly lobed. In Burkina Faso, kenaf leaves are used to prepare sauces. The flowers are 8–15 cm diameter, white, yellow or purple; when white or yellow, the center is still dark purple. The fruit is a 2-cm-diameter capsule containing several seeds. Because of their high mechanical strength, kenaf fibers are usually used in West Africa to manufacture ropes and sacks. In Burkina Faso, some local populations also use the fibers to make masks for traditional ceremonies.

In West Africa, before the introduction of industrial materials such as steel and concrete, adobes were traditionally stabilized or reinforced by locally available organic matter such as plant fibers, plant decoctions and cow dung. Many studies have dealt with the physical and mechanical characteristics of adobes stabilized or reinforced with natural fibers [1,2,3,4,5,6,7,8,9,10] but little attention has been paid to how the physicochemical characteristics of the incorporated fibers affect the physical and mechanical properties of the adobes elaborated. Moreover, although the chemical compositions of kenaf fibers from some countries such as Malaysia, Thailand, India, China, the southern United States, Mexico and Korea have been reported in the literature, this is not the case for kenaf fibers from Burkina Faso [11,12,13] and it is well known that the chemical composition of these fibers is mainly linked to the climate, plant species and type of soil.

The aim of this work is to study the influence of the physicochemical and mechanical characteristics of kenaf fibers from Burkina Faso on the mechanical behavior of adobes reinforced with these fibers. The final main objective is to valorize the fibers of the Kenaf plant in the building materials sector as it is easily produced in Burkina Faso, where it is abundant and cheap.

## 2. Materials and Testing Procedures

### 2.1. Raw Materials

**Figure 1:**
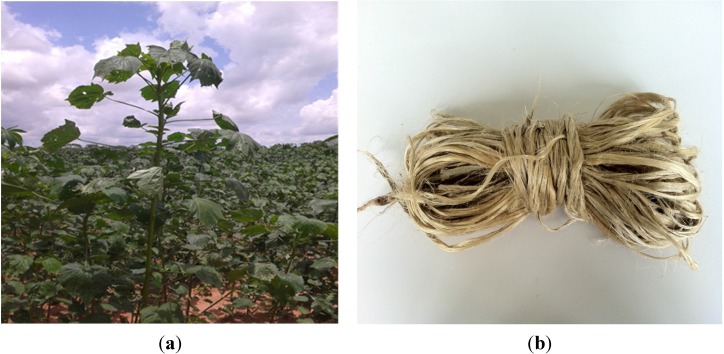
Pictures of kenaf plant (**a**) and extracted fibers (**b**).

The kenaf plant fibers were extracted near Bobo-Dioulasso (village of Farakoba) in the west of Burkina Faso. The extraction was done by hand, by pulling the fibers from the plant stalk. The Kenaf plant and extracted fibers are presented in Figure 1. The raw material used to manufacture adobes was a clayey soil from Rochechinard, a site located in the Isère valley (France). Its geotechnical characteristics have been published in a previous paper [14]. This raw material is composed of kaolinite (45 wt%), quartz (23 wt%), illite (14 wt%), goethite (7 wt%) and calcite (4 wt%) [15].

### 2.2. Procedures

#### 2.2.1. Physicochemical, Mineralogical and Mechanical Characteristics of Fibers

The fibers were considered as circular and their diameter was measured using a caliper with a precision of 0.01 mm. The natural humidity of the fibers was determined by drying fresh fibers in an oven at 105 °C for 24 h. The water absorption (W) was calculated on fibers soaked drinking water for 24 h [16].

To assess the mineralogical composition of the fibers, X-ray diffraction, thermal gravimetric analysis and Fourier transform infrared (FTIR) spectroscopy were performed on a crushed sample (size < 80 μm).

The thermal gravimetric analysis of the sample was performed at a constant heating rate of 10 °C/min. The X-ray diffraction apparatus used was a Siemens D5000 power X-ray Diffractometer equipped with a monochromator using a Kα (λ = 1.789 Å) cobalt anticathode. The thermal gravimetric curve of the fibers was obtained with a Netzsch SATA 449 F3 Jupiter apparatus and the infrared spectrum was obtained with a Nicolet 510FT-IR spectrometer operating in the range 4000−400 cm^−1^. A JEOL 6380 LV scanning electron microscope (JEOL, Croissy Sur Seine, France) equipped with a backscattered electron (BSE) detector was used for SEM observations on the fibers and the study of the morphology of the fibers was completed using an area video microscope, Keyence VH-5911 (Keyence, Osaka, Japan).

The fibers for chemical analysis were reduced to powder in a miller. The experimental technique used was the Van Soest procedure using four extracted detergents as NDS (Neutral Detergent Soluble), NDF (Neutral Detergent Fiber), ADF (Acid Detergent Fiber) and ADL (Acid Detergent Lignin) in order to quantify the amount of cellulose, hemicelluloses, lignin and ash of the fibers [17,18]. Cellulose is a linear polymer of β-(1-4)-D-glucopyranode. It exists in five types (I, II, III, IV and V). Type I is a native cellulose and has the best physical and mechanical properties (Young’s modulus of 150 GPa). Cellulose is a crystalline polysaccharide. Hemicellulose is composed of different types of saccharides such as xylose, mannose and glucose. It is strongly bound to the cellulose fibrils by hydrogen bonds [16]. Lignins are amorphous polymers formed by aromatic units such as guaiacycle, syringyl and phenylpropane. It acts as a cementing agent, binding the cellulose fibers together. 

The useful mechanical characteristics of the fibers are limited to their tensile behavior, which is classical for fibers (no shear and no compressive strength). The tensile behavior has already been described elsewhere [15].

#### 2.2.2. Preparation and Mechanical Characterization of Earth Blocks

**Figure 2:**
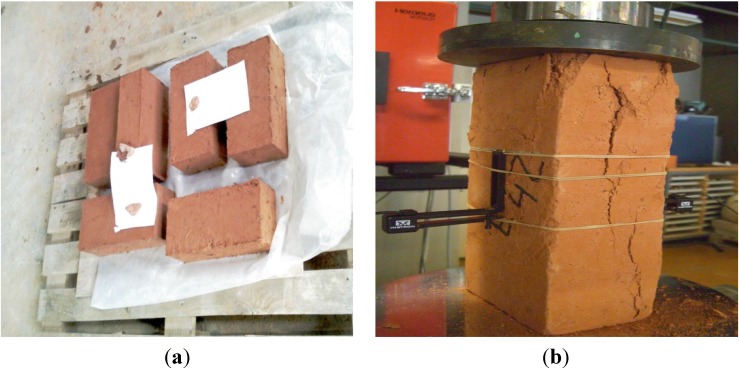
Compression test samples before test (**a**), sample after test with an extensometer in the central part of the sample measuring the strain (**b**).

Earth blocks were prepared using the technique of Pressed Adobe Blocks (PABs) [14]. The soil used for the manufacture of PABs was sieved to obtain particles with dimensions less than 5 mm. Then the prepared soil sample was mixed with 30-mm-long pieces of fiber for up to 0.8 wt% in relation to the dry weight of the soil. The average water/dry soil ratio used to manufacture the reinforced samples was 20 wt%. The soil composite was mixed for 15 min until it became a homogeneous paste. The different pastes were introduced into the rectangular prism mold (295 × 140 × 100 mm^3^) of a GEO 50 manual press to produce the pressed soil blocks. The pressure applied during compaction was approximately 2 MPa. The as-molded PABs were dried in the laboratory at room temperature (average 22 °C), with a humidity of 60% for an average period of 3 weeks until the block weight became constant. The unconfined compression test was performed on samples standing on their smallest faces in order to reduce the friction in the central part of the sample (Figure 2). It was very important for the test to be carried out in unconfined conditions in order to validate the assumption that the stress tensor was constant in the central part of the sample. This assumption enabled the stress to be calculated from the force measured by the press sensor. The details of the procedure are given elsewhere [19,20,21].

Extensometers placed in the central part of the sample measured the strain necessary for the calculation of Young’s modulus. It is now well known that earth materials have a very small elastic domain and that plasticity begins before 20% of the compressive strength [22,23]. As it was difficult to assess small strains at the beginning of the test, it was decided to work in cycles to measure the elasticity modulus. The procedure is not a standard one but details are given elsewhere [19,20,21,22,23].

The three-point bending strength tests were carried out using an INSTRON hydraulic press on the PAB specimens. The test was conducted at a constant speed of 0.01 mm·s^−1^. The load sensor used had a capacity of 50 kN. The mechanical tests have been carried out following the procedures given in previous papers [24,25].

## 3. Results and Discussion

### 3.1. Physicochemical, Mineralogical and Mechanical Characteristics of Fibers

**Figure 3:**
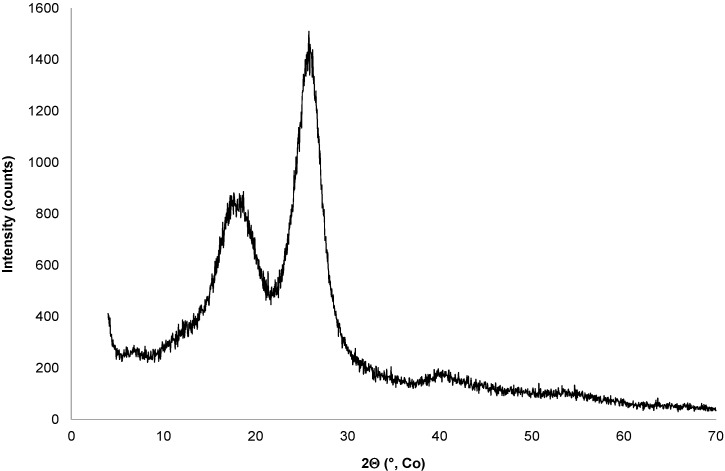
X-ray diffraction pattern of the fibers.

The X-ray diffraction pattern of crushed fibers is presented in Figure 3.

This pattern shows the presence of crystallized native cellulose with broad peaks located at 17.68°, 18.68°, 25.8°, 39.84° and 40.74°. The peaks at 17.68° and 18.68° are individualized in the X-ray diffraction pattern. This result indicates a significant amount of cellulose I in the studied fibers and is the same as those obtained with kenaf fibers from Malaysia and Korea [11,12]. The main peak at 39.84° in 2θ is not a doublet; it proves that the cellulose contained in the above fibers is cellulose I and this confirms its high Young’s modulus. In order to study the crystallinity of the cellulose contained in the fibers, the crystalline index of cellulose (I_c_) was evaluated using Segal’s method with the equation: Ic = (I_002_-I_am_)/I_002_ × 100 where I_002_ is the intensity of the XRD peak at 2θ = 25.8° and I_am_ the peak at 2θ = 18.68° [12]. The value obtained was 43. This low value is in the range of values reported in another paper on kenaf fibers [12] and is linked to the presence of amorphous compounds such as hemicelluloses and lignin in the raw fibers, which decrease the crystalline index.

**Figure 4:**
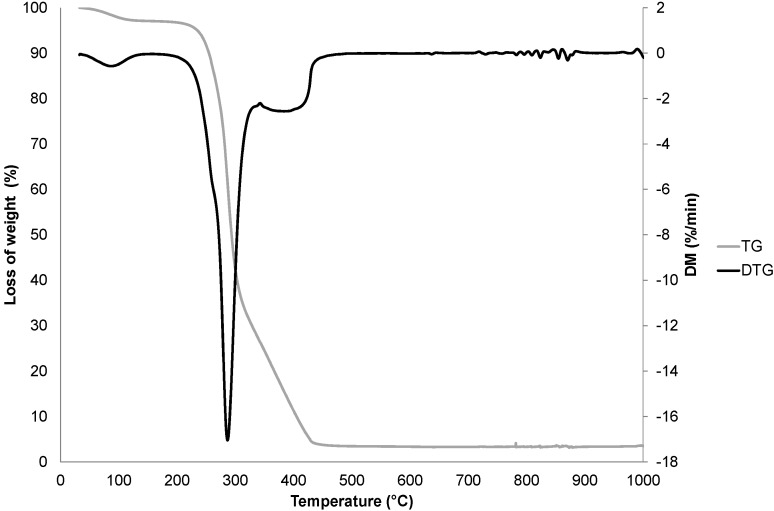
TG and DTG curves of the fibers.

To complete the mineralogical composition, and especially the characterization of amorphous compounds, the crushed fibers were analyzed by thermal gravimetric analysis (TGA) and Fourier transform infrared (FTIR) spectroscopy. Figure 4 shows the TGA curves for the sample.

The thermal characterization of the fibers during their sintering was carried out as described in the works of El-Shekeil *et al.* [26] and Morān *et al.* [27]. The first mass loss step around 100 °C was due to water loss from the fibers. The small mass loss step around 280 °C is a shoulder expressing the decomposition of thermally unstable compounds such as non-structural hemicelluloses and concerns the depolymerization of these hemicelluloses. This peak could also express the degradation of lignin. The major decomposition peak around 300 °C was attributed to cellulose decomposition. This large mass loss indicated that cellulose was a major compound of the fibers. The non-negligible peak around 400 °C is probably attributable to the structural hemicelluloses, lignin and cellulose which were pyrolyzed at this temperature. The attribution of this peak to lignin decomposition is supported by the fact that the lignin macromolecule is heat resistant. Lignin is thermally stable because, in its molecular structure, there is the possibility to form hydrogen bonds, which are known to stabilize the molecules. The thermal phenomenon could also be due to oxidative degradation of the charred residue.

**Figure 5:**
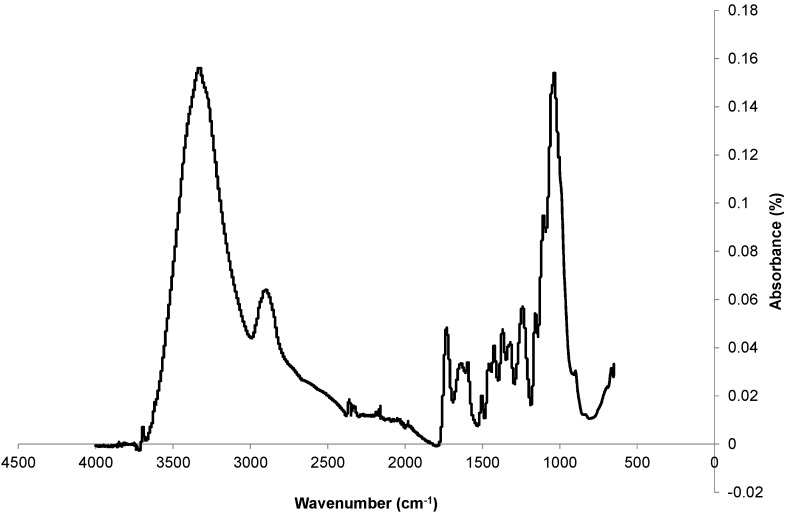
Infrared spectrum of the fibers.

The FTIR spectrum of the fibers is presented in Figure 5.

The interpretations of this spectrum were supported by the works of Ivanova *et al.* [28], Morān *et al.* [27] and Shin *et al.* [11]. The broad band around 3400 cm^−1^ corresponds to the stretching of the O-H bond of cellulose molecules. This band also characterizes the stretching of the O-H bond of water absorbed by fibers. The spectrum exhibits C-H stretching around 2900 cm^−1^. The peak at 1732 cm^−1^ is associated with C=O stretching of the acetyl group in hemicelluloses. This peak can be attributed to the p-coumeric acids of lignin or hemicelluloses. It could also express the presence of pectin in the fibers. The presence of proteins in the fibers is proved by the 1633 cm^−1^ and 1596 cm^−1^ peaks, which characterize the presence of primary and secondary amides. The peak at 1242 cm^−1^ is due to the C-O stretching of the aryl group in lignin. In the frequency range 800–1200 cm^−1^, valence vibrations of C-O, C-C and ring structures and external deformational vibrations of CH_2_, COH, CCO and CCH groups of cellulose are visible.

**Figure 6:**
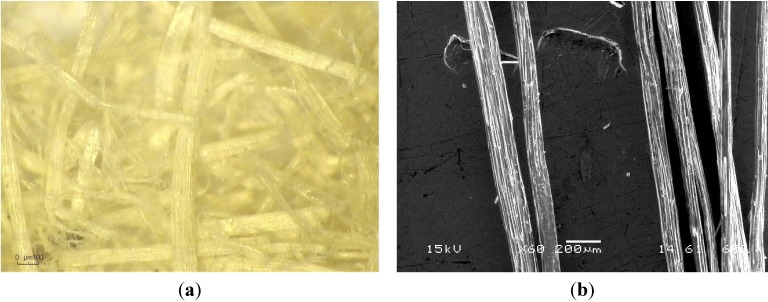
Video microscope image (**a**) ×175 and SEM micrograph (**b**) ×60 of the fibers.

The video-microscopy image and the SEM micrograph of the fibers are presented in Figure 6.

The observation of kenaf fibers reveals that they are arranged in beams with a rough surface that would be favorable for adherence with the earth in reinforced adobes. The fiber surfaces are covered by veins and they are surrounded by dark residues which could be pieces of the bark of the plant. The mean estimated diameter obtained with 20 isolated fibers was 60 μm. This value is within the range obtained by measurement with a micrometer. The fibers observed by SEM show a streaked structure with grooves. The isolated fibers are oriented in a single direction. The fiber surfaces are covered with dispersed impurities. According to the work of Jonoobi *et al.* on kenaf fibers [12], these impurities are hemicelluloses, lignin, pectin and waxy substances.

The chemical composition of the studied fibers and other kenaf fibers is presented in Table 1.

**Table 1:** Chemical composition of various kenaf fibers.

Reference	Cellulose (wt%)	Hemicelluloses (wt%)	Lignin (wt%)	Ash (wt%)
Present Study	70	19	3	1.3
[29]	53 ± 4	18 ± 1.4	8 ± 1.2	-
[13]	45–57	21.5	8–13	-
[12]	58 ± 1	22 ± 1	17.5 ± 1.3	2.4 ± 0.4
[11]	60.8	19.2	14.7	-
[30]	31–39	21.5	15–19	-

The cellulose, hemicelluloses and lignin contents diverge for different kenaf fibers. The cellulose content of the fibers studied here is greater than that of the other kenaf fibers. This disparity may be due to the climate, soil nature and the species of the plant. The hemicelluloses content is in the same range of values, whereas the lignin content is smaller than in any other study.

Table 2 presents the chemical composition of some other natural fibers for comparison with the kenaf fibers.

**Table 2:** Chemical composition of the fibers studied here compared to other types of fibers [9,13,28,29].

Reference	Cellulose (wt%)	Hemicelluloses (wt%)	Lignin (wt%)
Fibers studied here	70	19	3
Flax	71	19–20.6	2.2
Hemp	70–74	18–22.4	3.7–5.7
Jute	61–71	14–20	12–13
Ramie	68–76	13–17	0.6–0.7
Sisal	63–64	12.0	10–14
Banana	63–64	10	5
Cotton	85–90	5.7	-
Coir	32–43	0.15–0.25	40–45
Cereal straw	38–45	15–31	12–20
OPEFB * fibers	59	2.1	25
Groundnut shell	36	19	30
Bagasse	40–46	24.5–29	12.5–20
Rice husk	31	24	14
Coconut coir	47.7	25.9	17.8

Note: * Oil Palm Empty Fruit Bunch.

The discussion is focused on the cellulose content, which has the most influence on the mechanical characteristics of fibers because of its high tensile strength. The cellulose content of the fibers shown above is almost the same for flax, hemp (a variety of *cannabinus*), jute and ramie. The cellulose content of kenaf fibers is greater than that of the fibers of banana, cereal straw, oil palm empty fruit bunch, sisal, groundnut shell, rice husk, bagasse and coconut coir but cotton plant fibers are richer in cellulose than kenaf [30]. The fact that kenaf fibers have better mechanical properties than sisal, coir, coconut and oil palm empty fruit bunch is explained by their cellulose content.

The physical and mechanical properties of the fibers studied in the present work (diameter, natural humidity, specific weight, water absorption, specific weight, elasticity modulus and tensile strength) are given in Table 3. The diameters and the specific weights of kenaf fibers were in the same range as values for sisal, coconut and Lechuguilla fibers [2,8] but were smaller than those reported for straw fibers and oil palm empty fruit bunch fibers [5,9]. The natural humidity of the fibers was lower but their water absorption was higher than that of sisal, coconut and Lechuguilla fibers [2,8]. Straw fibers presented 500%–600% higher water absorption than kenaf fibers [5].

The tensile strength had a mean value of 1 GPa, with a high standard deviation (around 0.25 GPa) depending on the natural variability of the fibers. The tensile strength of kenaf fibers was higher than the tensile strength of sisal, lechuguilla, coconut, coir and oil palm empty fruit bunch fibers [2,8,9,13]. The higher cellulose content of the kenaf from Burkina Faso (Table 2) may be the reason why the mechanical characteristics of the fibers are higher than those of fibers of other origins. It should not be forgotten that, in the context of local materials, it is always necessary to test the materials to assess their performance [19], because of the variability of their composition depending on the soil, climate and plant species.

**Table 3:** Physical and mechanical properties of Kenaf fibers.

Properties	Results
Diameter (mm)	0.13
Natural humidity: H (%)	6.1
Water absorption: W (%)	307
Specific weight: γ (g/cm^3^)	1.04
Elasticity modulus (MPa)	136 ± 25
Tensile strength: σ (GPa)	1 ± 0.25

The elasticity modulus of kenaf was close to that of native cellulose (type I) and cellulose I could, therefore, be a major component of the fibers studied. The tensile strength was approximately twice that of steel with half the stiffness. These results reveal that kenaf fibers are 4 times more deformable than steel. This deformability is favorable when the fibers are used to reinforce soil for the production of PAB, which is a material with a stiffness approximately 200 times less than steel [14]. This point will be explored in the next section.

### 3.2. Mechanical Properties of Earth Blocks

**Figure 7:**
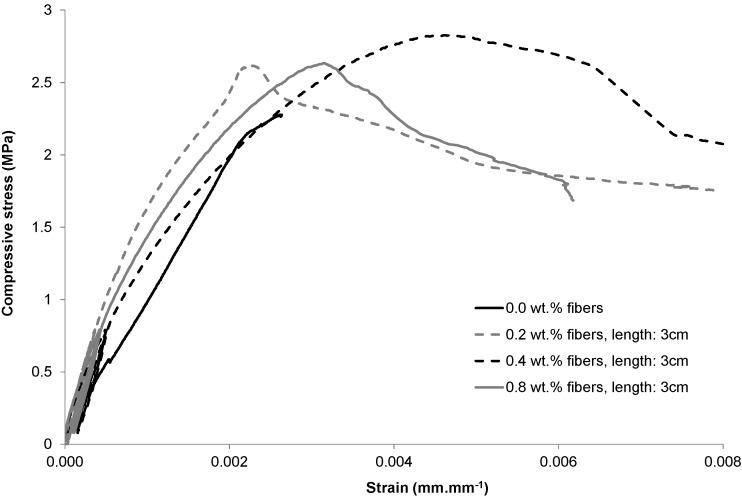
Compressive stress-strain behavior of fiber-reinforced PABs.

To highlight the behavior of PABs within a masonry structure, a stress-strain study was carried out using extensometers in unconfined compression tests with three cycles of loading and unloading (Figure 7). As observed, compressive strength increased with fibers additions. This result is due to the fact that fiber incorporation in the blocks prevents the propagation of cracks through their good adherence to the clay matrix because of their rough surface.

In a previous paper [15], two fiber lengths were tested. It was shown that the 3 cm length was more effective. Therefore only 3-cm-long fibers are considered here. The compressive strength increased to an optimum value near 0.4 wt% and then decreased. This is a classical result in soils reinforced by fiber [30]. At this optimum, the compressive strength increase was 16%. This increase in the compressive strength is small but, in general, the compressive strength of earth blocks is sufficient for buildings a few stories high. What is important is to check that the addition of fibers does not decrease the compressive strength (this is discussed in detail elsewhere [15]) as the addition of fibers may weaken the clay matrix by increasing the void content [30].

**Figure 8:**
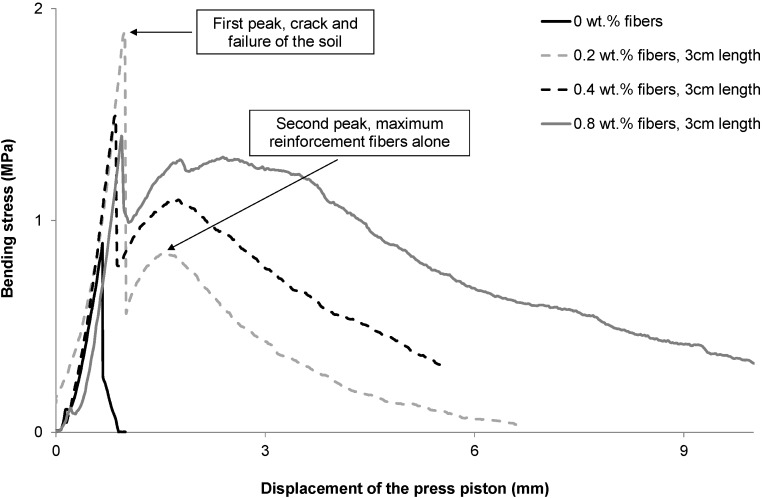
Flexural stress-train behavior of fiber-reinforced PABs, the span length is 28 cm.

Fibers are added to earth for two main reasons: to limit shrinkage cracks during the drying process of the material after manufacture of the blocks, and to improve their tensile ductility. That is why it is necessary to conduct both bending and tensile tests. Figure 8 presents the flexural behavior of fiber-reinforced PABs.

As it is shown in Figure 8, fibers incorporation increased the tensile strength of the PABs. This result is due to the good adherence of fibers with clay matrix and mainly their high tensile strength linked to their high content of cellulose (70 wt%), known for its high tensile strength (average 500 MPa). The importance of fiber incorporation is that they reinforce the blocks after the failure of the soil matrix, increasing the ductility of PABs.

The tensile behavior of unreinforced PABs shows only one stress peak, whereas those of PABs reinforced by fibers show two stress peaks [4]. The first stress peak is attributed to the cracking of the clay matrix of the PABs for all samples (reinforced and unreinforced). From the first peak, the residual stress is due to the fibers crossing the cracks that appear with the first peak. This is the new ductile behavior provided solely by the fibers crossing the crack. The area beneath the curves gives the ductility gained by adding fibers. This area is approximately proportional to the fiber content. It can be seen that, whether the fiber content is smaller than or equal to 0.8 wt%, the embedded length of the fibers crossing the crack is enough to mobilize most of the fibers’ tensile strength.

However, adding more fibers than 0.8 wt% may weaken the soil matrix and the friction mobilized by the embedded length of the fibers crossing the cracks may decrease, becoming too small to anchor the fibers. The fibers would be pulled out and their tensile strength would not be mobilized.

The second peak is specific to reinforced PABs and gives the maximum average strength of the fibers. To explain this phenomenon, two types of fibers crossing the crack must be considered separately. Let L_me_ being the minimum embedded length necessary to mobilize enough friction to reach the tensile strength of the fibers and let L_e_ be the embedded length of a fiber (the smallest length from either side of the crack).

In the Figure 8, for a given displacement the following can be set:
Case L_e_ < L_me_:(a) fibers with decreasing tensile stress. The fiber is pulled out and its embedded length is decreasing.(b) Fibers with an increasing tensile stress; the embedded length of these fibers is greater than the fibers in case (a).Case L_e_ < L_me_:(c) Broken fibers; the reinforcement is zero.(d) Fibers with an increasing tensile stress, the embedded length of these fibers is smaller than the fibers in case (c).


As the fibers are randomly distributed, the balance between all four types of fibers results in a decrease in strength after the second peak. In tensile tests the unreinforced PABs are brittle, whereas those reinforced with fibers have more ductile behavior, and this is very important for masonries, which usually show brittle behaviour. More ductile behavior of the blocks and mortar makes the masonry more stable against earthquakes and differential settlements, limiting crack growth.

In conclusion, the increased ductility of reinforced samples is linked to fibers that hold the cracks together after the failure of the clay matrix. The tensile strength of the fibers crossing the cracks is mobilized thanks to their embedded length, where a bond is created essentially between cellulose molecules (negative charge of O-H bonds) and, for example, flocculated cations such as Fe^3+^, Ca^2+^, and Mg^2+^ in the soil.

## 4. Conclusions

The physicochemical and mechanical characteristics of kenaf fibers from Burkina Faso and the mechanical properties of PABs reinforced with these fibers were investigated in this work. The conclusions drawn can be summarized as follows:

1) Kenaf fibers have a tensile strength higher than 750 MPa with a high elasticity modulus (136 ± 25 GPa) compared to other natural fibers.
2) Kenaf fibers contain large amounts (70 wt%) of cellulose type I (I_c_ = 40), with hemicelluloses (19 wt%) and lignin (3 wt%).
3) The incorporation of kenaf fibers in PABs improves mainly the ductility in tension of the blocks thanks to the high mechanical strength of fibers and their strong adherence to the clay matrix.
4) The incorporation of fibers is particularly beneficial as far as the bending strength of earth blocks is concerned.
5) In order to obtain optimum mechanical behaviour, an amount smaller than 0.8 wt% of short fibers (30 mm) is recommended.

## References

[1] Binici H., Aksogan O., Shah T. (2005). Investigation of fibre reinforced mud bricks as a building material. Constr. Build. Mater..

[2] Ghavami K., Toledo Filho R.D., Barbosa N.P. (1999). Behaviour of composite soil reinforced with natural fibres. Cem. Concr. Compos..

[3] Toledo Filho R.D., Ghavami K., England G.L., Scrivener K. (2003). Development of vegetable fibre-mortar composites of improved durability. Cem. Concr. Compos..

[4] Mesbah A., Morel J.C., Walker P., Ghavami K. (2004). Development of a direct tensile test for compacted soil blocks reinforced with natural fibers. J. Mater. Civil Eng..

[5] Bouhicha M., Aouissi F., Kenai S. (2005). Performance of composite soil reinforced with barley straw. Cem. Concr. Compos..

[6] Kumar A., Walia S.B., Mohan J. (2006). Compressive strength of fiber reinforced highly compressible clay. Constr. Build. Mater..

[7] Yetgin S., Cavdar O., Cavdar A. (2008). The effects of the fiber contents on the mechanic properties of the adobes. Constr. Build. Mater..

[8] Juárez C., Guevara B., Durán-Herrera A. (2010). Mechanical properties of natural fibers reinforced sustainable masonry. Constr. Build. Mater..

[9] Ismail S., Yaacob Z. (2011). Properties of laterite bricks reinforced with oil palm empty fruit bunch fibres. Pertanika J. Sci. Technol..

[10] Quagliarini E., Lenci S. (2010). The influence of natural stabilizers and natural fibres on the mechanical properties of ancient Roman adobe bricks. J. Cult. Herit..

[11] Shin H.K., Jeun J.P., Kim H.B., Kang P.H. (2012). Isolation of cellulose fibers from kenaf using electron beam. Radiat. Phys. Chem..

[12] Jonoobi M., Harun J., Tahir P.M., Shakeri A., SaifulAzry S., Makinejad M.D. (2011). Physicochemical characterization of pulp and nanofibers from kenaf stem. Mater. Lett..

[13] Akil H.M., Omar M.F., Mazuki A.A.M., Safiee S., Ishak Z.A.M., Abu Bakar A. (2011). Kenaf fiber reinforced composites: A review. Mater. Des..

[14] Kouakou H., Morel J.C. (2009). Strength and elasto-plastic properties of non-industrial building materials manufactured with clay as a natural binder. Appl. Clay Sci..

[15] Millogo Y., Morel J.C., Aubert J.E., Ghavami K. (2014). Experimental analysis of pressed adobe blocks reinforced with *Hibiscus cannabinus* fibers. Constr. Build. Mater..

[16] Toledo Filho R.D. (1997). Natural Fibre Reinforced Mortar Composites: Experimental, Characterisation. Ph.D. Thesis.

[17] Van Soest P.D., Wine R.H. (1967). The use of detergents in the analysis of fibrous feed II. A rapid method for determination of fiber and lignin. J. Assoc. Off. Agric. Chem..

[18] Fulgencio S.C., Jaime C., Juan G.R. (1983). Determination of hemicelluloses, cellulose, and lignin contents of dietary fibre and crude fiber of several seed hulls. Data Comp..

[19] Ciblac T., Morel J.C. (2014). Sustainable Masonry: Stability and Behavior of Structures.

[20] Aubert J.E., Maillard P., Morel J.C., Al Rafii M. (2015). Towards a simple compressive strength test for earth bricks?. Mater. Struct..

[21] Aubert J.E., Fabbri A., Morel J.C., Maillard P. (2013). A soil block with a compressive strength higher than 45 MPa!. Constr. Build. Mater..

[22] Olivier M., Mesbah A. Modèle de comportement pour sols compactés. Proceedings of the First International Conference on unsaturated soils.

[23] Bui Q.B., Morel J.C., Hans S., Walker P. (2014). Effect of moisture content on the mechanical characteristics of rammed earth. Constr. Build. Mater..

[24] Morel J.C., Pkla A. (2002). A model to measure compressive strength of Compressed Earth Blocks with the “3 points bending test”. Constr. Build. Mater..

[25] Morel J.C., Pkla A., di Benedetto H. (2003). Interprétation en compression ou traction de l’essai de flexion en trois points. Rev. Fr. Génie Civil.

[26] El-Shekeil Y.A., Sapuan S.M., Abdan K., Zainudin E.S. (2012). Influence of fiber content on the mechanical and thermal properties of Kenaf fiber reinforced thermoplastic polyurethane composites. Mater. Des..

[27] Morān J.I., Alvarez V.A., Cyras V.P., Vāsquez A. (2008). Extraction of cellulose and preparation of nanocellulose. Cellulose.

[28] Ivanova N.V., Korolenko E.A., Korolik E.V., Zhbankov R.G. (1989). IR spectrum of cellulose. J. Appl. Spectrosc..

[29] Godin B., Ghysel F., Agneessens R., Schmit T., Gofflot S., Lamaudière S., Sinnaeve G., Goffart J.P., Gerin P.A., Stilmant D. (2010). Détermination de la cellulose, des hemicelluloses, de la lignine et des cendres dans diverses cultures lignocellulosiques dédiées à la production de bioéthanol de deuxième génération. Biotechnol. Agron. Soc. Environ..

[30] Morel J.C., Gourc J.P. (1997). Behavior of sand reinforced with mesh elements. Geosynth. Int..

